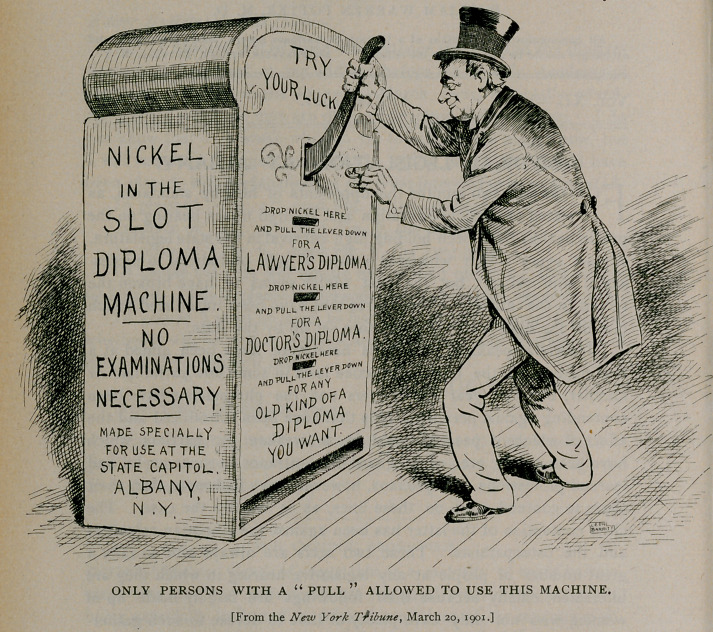# Medical Legislation at Albany

**Published:** 1901-04

**Authors:** 


A Monthly Review of Medicine and Surgery.

*EDITOR:*

WILLIAM WARREN POTTER, M. D.

All communications, whether of a literary or business nature, books for review and exchanges should be addressed to the editor: 284 FRANKLIN STREET, BUFFALO, N. Y.

VOL. XL.—LVI. APRIL, 1901. NO. 9.

### MEDICAL LEGISLATION AT ALBANY.

FOR the past two months the opponents of healthy standards, as related to medical education and kindred topics bearing on the public health, have been holding high carnival in the state legislature. Almost every winter witnesses a crop of bills prejudicial to professional improvement, but the past winter seems to have broken the record in the number of measures that have been introduced which menace the high standard heretofore set up by the state.

Generally speaking, such bills heretofore have been aimed at a modification of the medical practice laws in some one particular or another, but this year the lawyers and the pharmacists have been contending against pernicious bills that have been introduced and the effect of which, if passed, would be to weaken the barriers which have been wisely erected to guard the entrance to those useful and learned professions. Physicians, however, have had more than their share to contend with on these lines, as is usually the case. The chief contention of the latter has been against the Christian scientists and the osteopathists. These two sects are always able to rally a great number of people at any legislative hearing in which they are interested, chiefly because their followings are largely made up of women, who not only have plenty of time to devote to such gatherings, but who also have a penchant for the dramatic, which always develops in a greater or less degree on those occasions.

On the other hand physicans, whose time is taken up in serving the sick, generally find it difficult to leave home on a sudden call of this kind, hence are sparingly represented in numbers before committees that grant public hearings. The feeling has undoubtedly been intensified during the present legislative session, owing to the pernicious character of some of the bills that have been introduced. Indeed, it has been given out from Albany, we know not upon what authority, that the legislature, or some of the members thereof, has grown tired of the doctors (?) Small wonder, when we consider the fact that members of the legislature still can be found who will lend themselves to the introduction of measures that are calculated not only to

ONLY PERSONS WITH A "PULL" ALLOWED TO USE THIS MACHINE.

[From the *New York Tribune*, March 20, 1901.]

irritate the profession of medicine, but to exasperate the friends of medical education and of public health safeguards throughout the state.

We have more than once, not only in the columns of this journal, but on public occasions and in private conversation, urged the propriety of the legislature declining to consider bills relating to these matters unless they emanate from responsible bodies, representative in character, like the regents of the university or the state medical societies. For ten years and more the Medical Society of the State of New York appeared annually at the bar of the legislature, praying for advanced standards in medical education. The Homeopathic and Eclectic state medical societies finally joined in this crusade, which ultimately resulted in persuading the legislature to give us one of the best practice acts that any state has yet evolved.

Briefly summarised, it has established preliminaries equal to a high school diploma; it requires four years' training in a medical college before the degree of doctor of medicine can be conferred; and, finally, it requires a separate examination by the state, the details of which are duly prescribed by law, before license to practise can be obtained.

Scarcely had this new order of things been established in 1891, when attempts were begun to weaken or invalidate some one or all of the beneficent provisions just referred to; and for ten years past it has required the constant vigilance of the constituted medical authorities of the state to prevent or avert such direful disaster. Strangely enough, these attempts are made possible by the action of members of the legislature who introduce measures, received from irresponsible persons who have some personal interest to further, having little or no regard for the general good; but, on the other hand, who apparently nurse an especial hatred toward the medical practice act.

It would seem to be pretty nearly time, therefore, that a busy legislature like ours should rid itself of all such unnecessary and unwholesome measures, by referring them back to the representative medical societies or to the regents' office for opinion before taking definite action. We sympathise with our friends the lawyers and the pharmacists in the similar troubles they are experiencing, and would suggest a similar method of relief. When a plan of dealing with these measures, like the one suggested, can be adopted, then, and only then, will medical education in the Empire State be placed upon an enduring basis. It would not embarrass but rather relieve the individual member who is besought to introduce these measures. He could present them by request when he desired to do so; they could then, as now, be referred to the appropriate committees, and the chairmen of these could refer them to the regents or to medical, legal, or pharmaceutical societies, as the case might be, for an opinion as to their merits.

In this way the interested societies would get official notice of the measures, and unnecessary trips to Albany would be prevented. The present method of dealing with these questions is a scandal to the intelligence of the period, and a blotch on the escutcheon of parliamentary procedure.

## Figures and Tables

**Figure f1:**